# Supplementary figures and images for: In silico analyses identify sequence contamination thresholds for Nanopore-generated SARS-CoV-2 sequences
Source: PLoS Comput Biol. 2024 Aug 19;20(8):e1011539. doi: 10.1371/journal.pcbi.1011539 (PMC11398645; doi:10.1371/journal.pcbi.1011539)

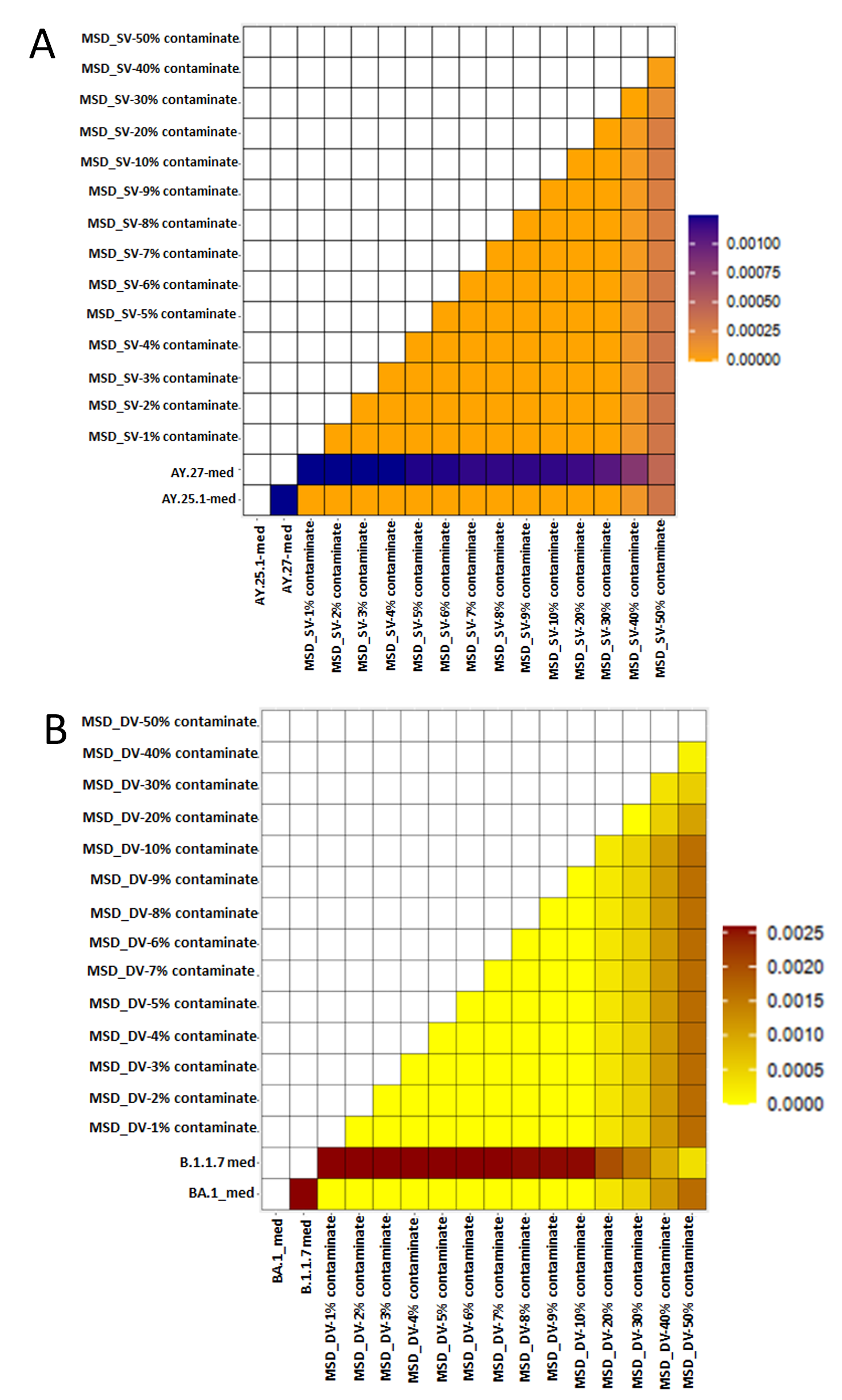

Supplement: S1 Fig — A) A heatmap of the pairwise p-distance comparison of the MSD_SV samples—a delta background sequence (AY.25.1) contaminated with a similar delta contaminant sequence (AY.27). B) A heatmap of the pairwise p-distance comparison of the MSD_DV samples–an omicron background sequence (BA.1) contaminated with an alpha contaminant sequence (B.1.1.7). (TIF) [file pcbi.1011539.s001.tif]

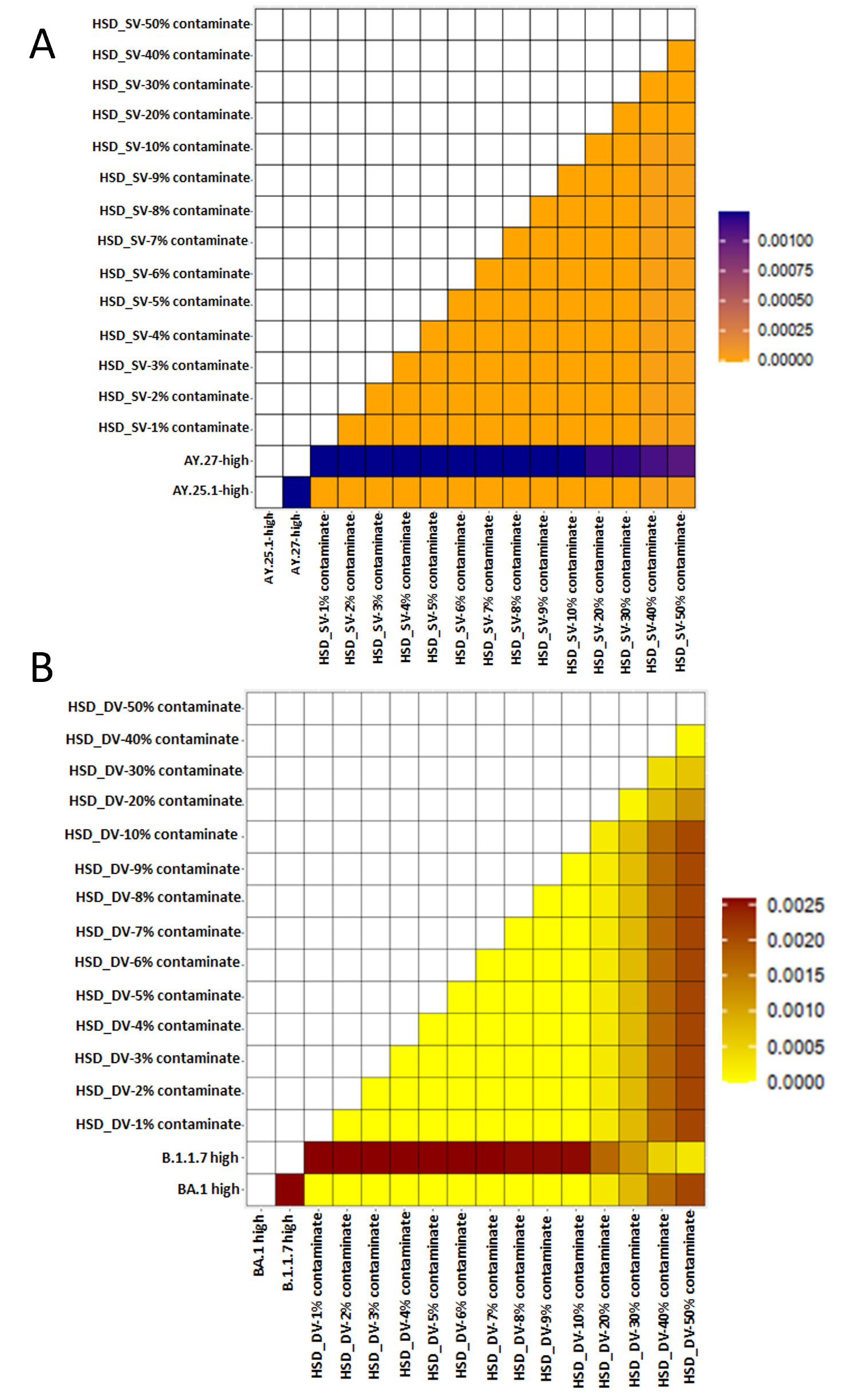

Supplement: S2 Fig — A) A heatmap of the pairwise p-distance comparison of the HSD_SV samples—a delta background sequence (AY.25.1) contaminated with a similar delta contaminant sequence (AY.27). B) A heatmap of the pairwise p-distance comparison of the HSD_DV samples–an omicron background sequence (BA.1) contaminated with an alpha contaminant sequence (B.1.1.7). (TIF) [file pcbi.1011539.s002.tif]

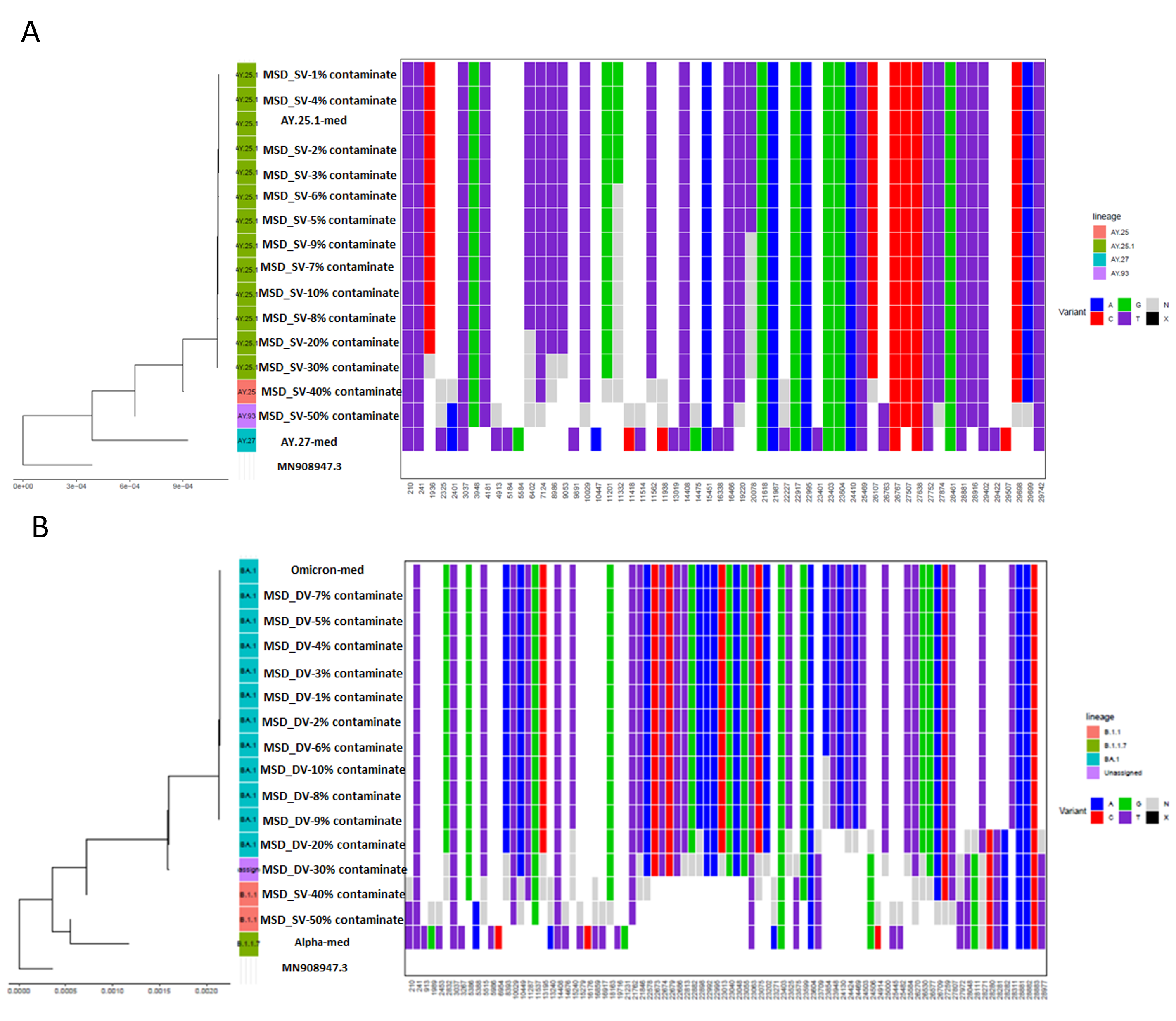

Supplement: S3 Fig — Phylogenetic tree and heatmaps showing single nucleotide variation at different positions of the SARS-CoV-2 genome for (A) a delta variant (AY.25.1) contaminated with another delta variant (AY.27) sequence at contamination levels 1–10%, 20%, 30%, 40%, and 50% for medium sequencing depth and (B) an omicron variant (BA1) contaminated with an alpha contaminant sequence (B.1.1.7) at contamination levels 1–10%, 20%, 30%, 40%, and 50% for medium sequencing depth (25,000 reads). (TIF) [file pcbi.1011539.s003.tif]

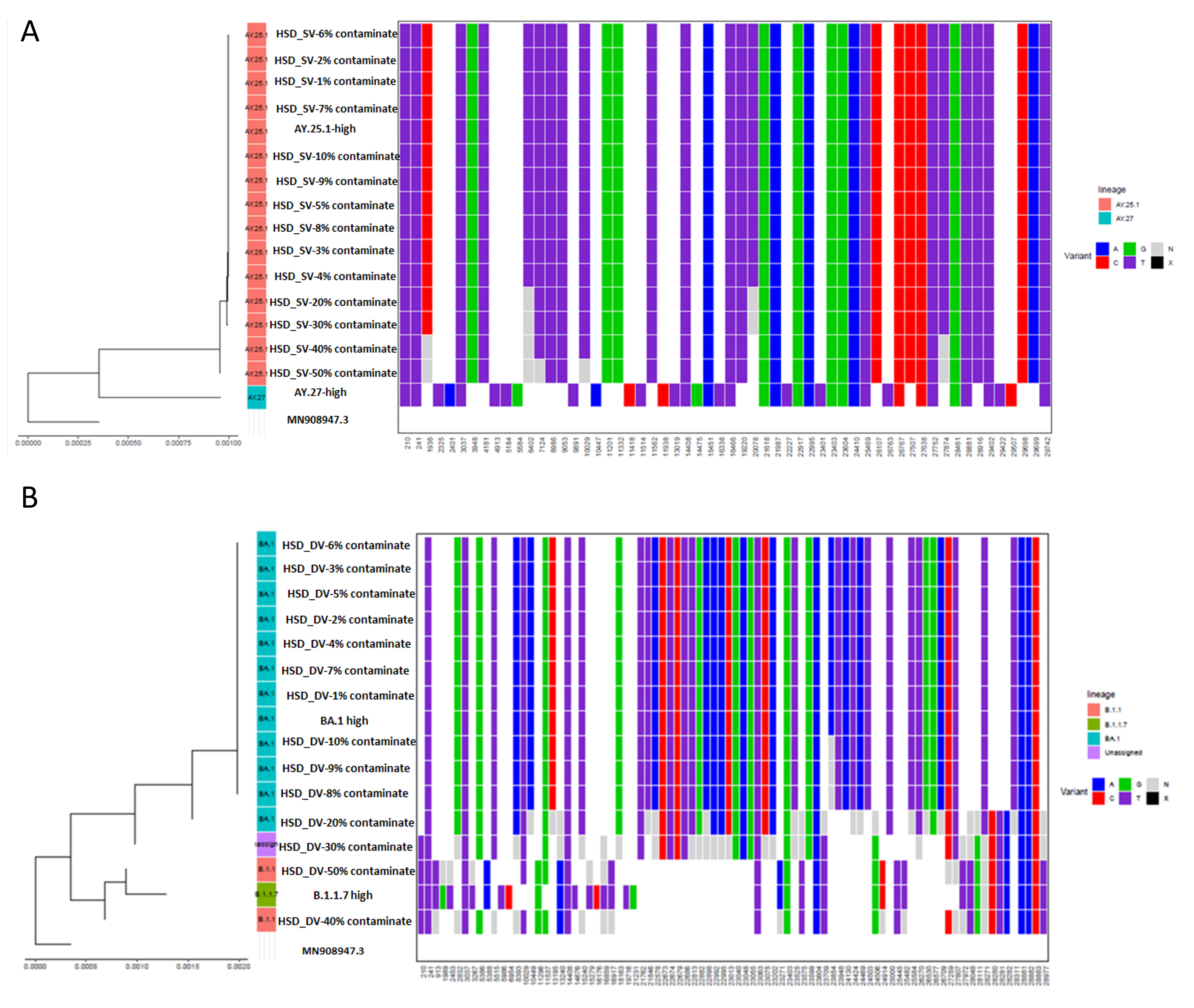

Supplement: S4 Fig — Phylogenetic tree and heatmaps showing single nucleotide variation at different positions of the SARS-CoV-2 genome for (A) a delta variant (AY.25.1) contaminated with another delta variant (AY.27) sequence at contamination levels 1–10%, 20%, 30%, 40%, and 50% for high sequencing depth and (B) an omicron variant (BA1) contaminated with an alpha contaminant sequence (B.1.1.7) at contamination levels 1–10%, 20%, 30%, 40%, and 50% for high sequencing depth (50,000 reads). (TIF) [file pcbi.1011539.s004.tif]

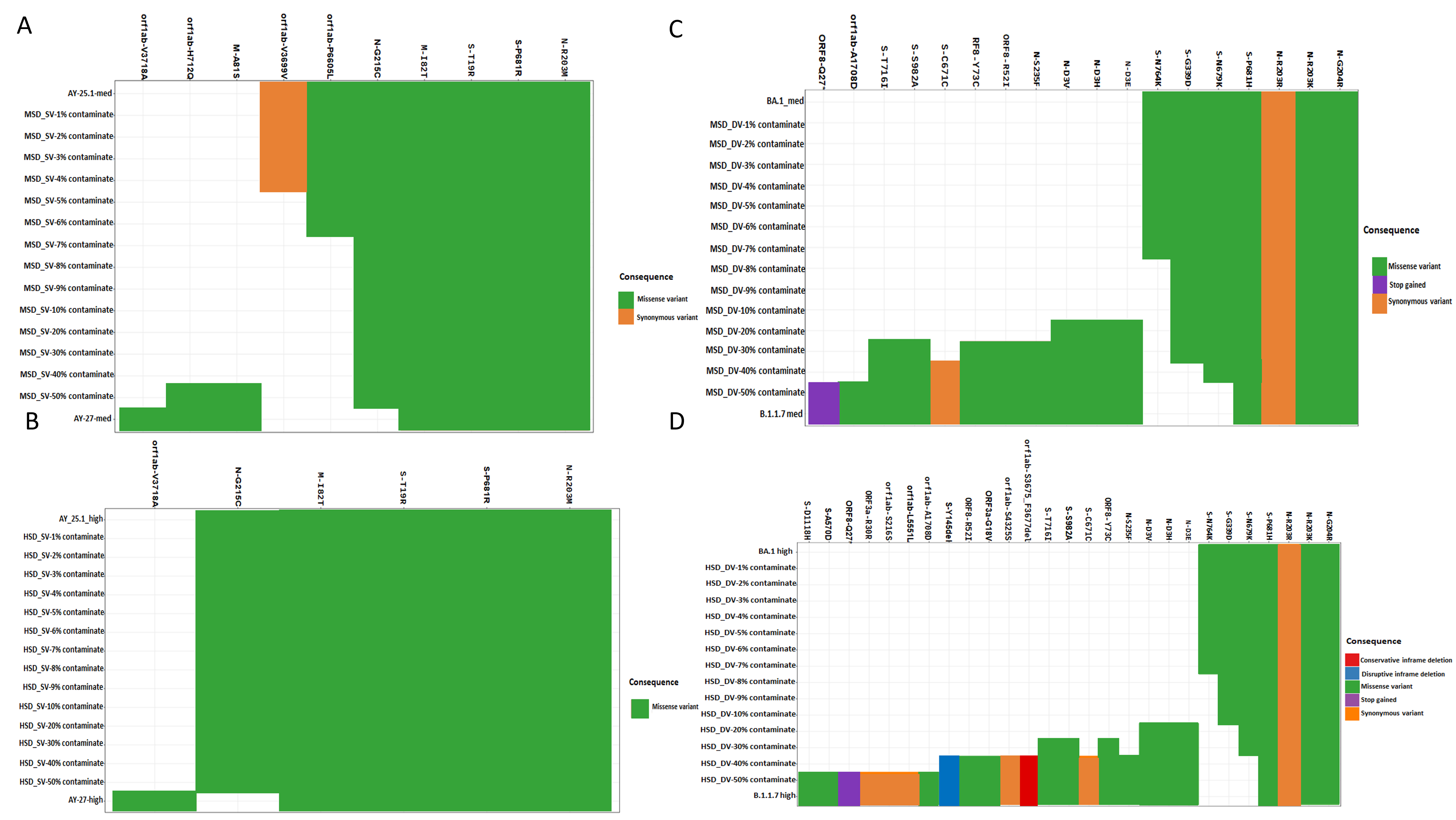

Supplement: S5 Fig — Mutational profile comparison of SARS-CoV-2 genome for the clinical genomes to the artificially generated genomes for (A) MSD_SV and (B) (AY.25.1 contaminated with an AY.27 variant) sequence at contamination levels 1–10%, 20%, 30%, 40%, and 50%. (C) MSD_DV and (D) HSD_DV (BA.1 contaminated with a B.1.1.29 variant) at contamination levels 1–10%, 20%, 30%, 40%, and 50%. (TIF) [file pcbi.1011539.s005.tif]
