# Supplementary material for: In silico analyses identify sequence contamination thresholds for Nanopore-generated SARS-CoV-2 sequences
Source: PLoS Comput Biol. 2024 Aug 19;20(8):e1011539. doi: 10.1371/journal.pcbi.1011539 (PMC11398645; doi:10.1371/journal.pcbi.1011539)
Supplement: S2 Table — A. Quality control metrics comparison for artificially subsampled and contaminated genomes of contamination by similar variants at a high sequencing depth–for all HSD_SV genomes. B. Quality control metrics comparison for artificially subsampled and contaminated genomes of contamination by different variants at a high sequencing depth–for all HSD_DV genomes. (DOCX) [file pcbi.1011539.s007.docx]

S2 Table. A

| Genome | **Num. of consensus_snvs** | **Number of consensus ‘N’** | **Number of variants SNVs** | **Number of variants indel** | **Mean sequencing depth** | **Genome completeness** | **Lineage** | **Scorpio calls** | **Watch mutations** |
| --- | --- | --- | --- | --- | --- | --- | --- | --- | --- |
| **AY.25.1 high** | 40 | 189 | 45 | 3 | 1887.9 | 0.9937 | AY.25.1 | Delta (B.1.617.2-like) | S:G142D,S:L452R |
| HSD_SV-1% contaminate | 40 | 189 | 45 | 3 | 1888 | 0.9937 | AY.25.1 | Delta (B.1.617.2-like) | S:G142D,S:L452R |
| HSD_SV-2% contaminate | 40 | 189 | 45 | 3 | 1888 | 0.9937 | AY.25.1 | Delta (B.1.617.2-like) | S:G142D,S:L452R |
| HSD_SV-3% contaminate | 40 | 189 | 45 | 3 | 1888 | 0.9937 | AY.25.1 | Delta (B.1.617.2-like) | S:G142D,S:L452R |
| HSD_SV-4% contaminate | 40 | 189 | 45 | 3 | 1888 | 0.9937 | AY.25.1 | Delta (B.1.617.2-like) | S:G142D,S:L452R |
| HSD_SV-5% contaminate | 40 | 189 | 45 | 3 | 1888 | 0.9937 | AY.25.1 | Delta (B.1.617.2-like) | S:G142D,S:L452R |
| HSD_SV-6% contaminate | 40 | 189 | 45 | 3 | 1888 | 0.9937 | AY.25.1 | Delta (B.1.617.2-like) | S:G142D,S:L452R |
| HSD_SV-7% contaminate | 40 | 189 | 45 | 3 | 1888 | 0.9937 | AY.25.1 | Delta (B.1.617.2-like) | S:G142D,S:L452R |
| HSD_SV-8% contaminate | 40 | 189 | 45 | 3 | 1888 | 0.9937 | AY.25.1 | Delta (B.1.617.2-like) | S:G142D,S:L452R |
| HSD_SV-9% contaminate | 40 | 189 | 45 | 3 | 1887.9 | 0.9937 | AY.25.1 | Delta (B.1.617.2-like) | S:G142D,S:L452R |
| HSD_SV-10% contaminate | 40 | 189 | 45 | 3 | 1887.9 | 0.9937 | AY.25.1 | Delta (B.1.617.2-like) | S:G142D,S:L452R |
| HSD_SV-20% contaminate | 38 | 191 | 43 | 3 | 1887.8 | 0.9936 | AY.25.1 | Delta (B.1.617.2-like) | S:G142D,S:L452R |
| HSD_SV-30% contaminate | 38 | 191 | 43 | 3 | 1887.9 | 0.9936 | AY.25.1 | Delta (B.1.617.2-like) | S:G142D,S:L452R |
| HSD_SV-40% contaminate | 36 | 192 | 41 | 3 | 1887.6 | 0.9936 | AY.25.1 | Delta (B.1.617.2-like) | S:G142D,S:L452R |
| HSD_SV-50% contaminate | 34 | 194 | 38 | 3 | 1887.3 | 0.9935 | AY.25.1 | Delta (B.1.617.2-like) | S:G142D,S:L452R |
| **AY.27 high** | 39 | 189 | 43 | 3 | 1886.8 | 0.9937 | AY.27 | Delta (B.1.617.2-like) | S:G142D,S:L452R |

S2 Table. B

| Genome | **Num. of consensus_snvs** | **Number of consensus ‘N’** | **Number of variants SNVs** | **Number of variants indel** | **Mean sequencing depth** | **Genome completeness** | **Lineage** | **Scorpio calls** | **Watch mutations** |
| --- | --- | --- | --- | --- | --- | --- | --- | --- | --- |
| **BA.1 high** | 55 | 189 | 61 | 7 | 1881.3 | 0.9937 | BA.1 | Omicron (BA.1-like) | S:del69-70,S:K417N,S:Q493R,S:N501Y,S:P681H,S:P681H |
| HSD_DV-1% contaminate | 55 | 189 | 61 | 7 | 1881.5 | 0.9937 | BA.1 | Omicron (BA.1-like) | S:del69-70,S:K417N,S:Q493R,S:N501Y,S:P681H,S:P681H |
| HSD_DV-2% contaminate | 55 | 189 | 61 | 7 | 1881.4 | 0.9937 | BA.1 | Omicron (BA.1-like) | S:del69-70,S:K417N,S:Q493R,S:N501Y,S:P681H,S:P681H |
| HSD_DV-3% contaminate | 55 | 189 | 61 | 7 | 1881.4 | 0.9937 | BA.1 | Omicron (BA.1-like) | S:del69-70,S:K417N,S:Q493R,S:N501Y,S:P681H,S:P681H |
| HSD_DV-4% contaminate | 55 | 189 | 61 | 7 | 1881.4 | 0.9937 | BA.1 | Omicron (BA.1-like) | S:del69-70,S:K417N,S:Q493R,S:N501Y,S:P681H,S:P681H |
| HSD_DV-5% contaminate | 55 | 189 | 61 | 7 | 1881.5 | 0.9937 | BA.1 | Omicron (BA.1-like) | S:del69-70,S:K417N,S:Q493R,S:N501Y,S:P681H,S:P681H |
| HSD_DV-6% contaminate | 55 | 189 | 61 | 7 | 1881.5 | 0.9937 | BA.1 | Omicron (BA.1-like) | S:del69-70,S:K417N,S:Q493R,S:N501Y,S:P681H,S:P681H |
| HSD_DV-7% contaminate | 55 | 189 | 61 | 7 | 1881.6 | 0.9937 | BA.1 | Omicron (BA.1-like) | S:del69-70,S:K417N,S:Q493R,S:N501Y,S:P681H,S:P681H |
| HSD_DV-8% contaminate | 54 | 190 | 60 | 7 | 1881.6 | 0.9936 | BA.1 | Omicron (BA.1-like) | S:del69-70,S:K417N,S:Q493R,S:N501Y,S:P681H,S:P681H |
| HSD_DV-9% contaminate | 54 | 190 | 60 | 7 | 1881.7 | 0.9936 | BA.1 | Omicron (BA.1-like) | S:del69-70,S:K417N,S:Q493R,S:N501Y,S:P681H,S:P681H |
| HSD_DV-10% contaminate | 54 | 190 | 60 | 7 | 1881.7 | 0.9936 | BA.1 | Omicron (BA.1-like) | S:del69-70,S:K417N,S:Q493R,S:N501Y,S:P681H,S:P681H |
| HSD_DV-20% contaminate | 42 | 208 | 47 | 5 | 1882.1 | 0.993 | BA.1 | Probable Omicron (BA.1-like) | S:del69-70,S:K417N,S:Q493R,S:N501Y,S:P681H,S:P681H |
| HSD_DV-30% contaminate | 31 | 220 | 35 | 2 | 1882.5 | 0.9926 | Unassigned | Probable Omicron (Unassigned) | S:del69-70,S:Q493R,S:N501Y,S:P681H,S:P681H,S:T716I,S:S982A |
| HSD_DV-40% contaminate | 26 | 201 | 29 | 4 | 1882.7 | 0.9933 | B.1.1 |  | S:del69-70,S:del144,S:N501Y,S:P681H,S:P681H,S:T716I,S:S982A |
| HSD_DV-50% contaminate | 31 | 196 | 34 | 4 | 1883.1 | 0.9934 | B.1.1 |  | S:del69-70,S:del144,S:N501Y,S:A570D,S:P681H,S:P681H,S:T716I,S:S982A,S:D1118H |
| **B.1.1.7 high** | 40 | 189 | 45 | 4 | 1885.6 | 0.9937 | B.1.1.7 | Alpha (B.1.1.7-like) | S:del69-70,S:del144,S:N501Y,S:A570D,S:P681H,S:P681H,S:T716I,S:S982A,S:D1118H |
