# Supplementary material for: In silico analyses identify sequence contamination thresholds for Nanopore-generated SARS-CoV-2 sequences
Source: PLoS Comput Biol. 2024 Aug 19;20(8):e1011539. doi: 10.1371/journal.pcbi.1011539 (PMC11398645; doi:10.1371/journal.pcbi.1011539)
Supplement: Attachment — Submitted filename: Response to reviewers.docx [file pcbi.1011539.s008.docx]

The points made by Reviewer 3 at the end of the review comments (those to the editors) should be resolved by this study. We quote them as follows: The sequence of each virus is different as differed in human fingerprints; now the question is whether these differences should be treated as contamination, mutation, new strain or new reference.

Reviewer's Responses to Questions

**Comments to the Authors:
Please note here if the review is uploaded as an attachment.**

Reviewer #1: In this work, Bolaji and Duggan simulate contaminated Nanopore datasets of SARS-CoV2 genomes at different contamination levels and sequencing depths to study the effect of sample contamination on genome integrity and lineage calls. The raw reads are real but were mixed up at the FASTQ file step. After assembly with the ARTIC pipeline (following the Nanopolish variant calling path), those were further processed with ncov-tools and Pangolin. All datasets have been made available by the authors on Zenodo.

Overall, the study was well designed (even if modest in ambition) and the reported interpretations are in line with the results. I have some concerns regarding the significance of the conclusions, as well as the length and clarity of the manuscript, but I did not identify any major flaw in the authors' work that would make the study unpublishable after revision.

* Scientific significance

1. The objective of the authors is to provide contamination level thresholds under which genome integrity and lineage calls would be unaffected (e.g., lines 414-415). Moreover, they study the matter at different sequencing depths, from one typical multiplexed experiment (50k reads per sample) down to 1/4 of such an experiment (12.5k reads). I see the point but, in real life, one does not know the level of contamination. Therefore it would be more useful to identify clues in raw read datasets and/or assembled genomes that would help detecting and pinpointing sample contamination in a real setup (lines 456-457). In that respect the authors' suggestion about negative controls makes sense and could be further developed (lines 447-451).

**Response: We appreciate Reviewer #1’s comments as they indicate that our purpose was unclear. Through modeling the effect of contamination on *in silico* contaminated libraries, we developed guidelines for contamination that are intended to be used to guide the interpretation of negative control. We have modified the text to state this intention more clearly (lines 377-404) and have addressed how contamination by multiple strains may be addressed by including negative controls at all stages in your sequencing experiment workflow (lines 377-392). We propose that after each experiment, researchers investigate the number of reads in all negative controls. If the number of reads in the negative controls exceeds the recommended threshold, then the reads present in the negative controls should be mapped to the reference genome to determine the origin of contamination**.

2. On a related note, the authors rightfully raise the issue of recombinant genomes, for example "deltacron" viruses (lines 430-434). It is true that, taken at face value, contaminated samples could yield pseudo-recombinant genomes in terms of hybrid combinations of variant-specific SNVs (and thus intermediate p-distances with background "pure" samples). Yet, the mutational profile along the consensus genome should be different between true recombinants and contaminated assemblies. Indeed (maybe naively speaking), I would expect recent recombinant genomes to be composed of two different halves, each one corresponding to a "donor" variant whereas contaminated assemblies would display a more homogeneous mixture of diagnostic nucleotides and a stronger propension to have ambiguous nucleotides ('N') at these variable positions. Maybe these points would gain to be discussed in the manuscript.

**Response: We have adjusted the text to emphasized that the reference genome used for all the analysis is the SARS-CoV-2 Wuhan strain. This is why the mutational profile of the consensus genomes differs between the true recombinants and the contaminated assemblies. All assemblies were mapped to the Wuhan strain, comparisons were made and conclusions were drawn from the observation. Without access to multiple independent clinical samples from the same patient, it is exceedingly difficult to distinguish contamination from co-infection with multiple strains. As Reviewer #1 points out, recombinant genomes are expected to be composed of distinct sections of parental genomes however, the proliferation of Omicron sublineages and Omicron-Omicron recombinants makes this task increasingly difficult to distinguish.**

3. While the experimental design is sound, it is also modest, in the sense that only four starting variants were considered as the basis for the whole study (Table 1 and lines 452-454). What would be the effects of using different variants? Can the issue be really reduced to "similar variants" vs "different variants" without consideration for other p-distances between variants? Owing to these issues, it is difficult to determine whether the reported thresholds are generalizable to other (real) situations. In the same line of thought (hence in the same comment), there is no statistical framework at all in the study. This leads to sentences that are probably devoid of an actual statistical significance, e.g., "a decrease in the number of SNVs (from 40 to 39) and an increase in the number of consensus ‘n’ (from 189 to 190) were observed" (lines 236-237). I think that these claims should either be avoided or be backed up by statistics.

**Response: We have addressed this concern by deleting the following “A decrease in the number of SNVs (from 40 to 39) and an increase in the number of consensus ‘n’ (from 189 to 190) were observed”. The decision to exclude the claim instead of performing a statistical test is because the authors feel that the sample size utilized in the study, as well as the nature of the comparison performed, does not necessitate a statistical test. No replicates were included due to the scope of the study.**

* Manuscript organization and length

4. The results are faithfully reported but the study structure makes its reporting quite repetitive. There are similar blocks of text (and figures and tables) for each combination of sequencing depth (LSD, MSD, HSD) and p-distance (SV, DV) and even repetitions within a single combination, e.g., "In conclusion, for contamination by a similar SARS-CoV-2 variant, the contamination threshold identified for lineage call was 30% for both LSD_SV and MSD_SV and 50% for HSD_SV genomes. However, for genome integrity, the contamination threshold was 5% for low, 4% for medium, and 10% for high sequencing depths (Figures 4 A & B)" (lines 242-246). My opinion is that the net amount of knowledge brought by the study does not warrant such a long and repetitive manuscript. While it would totally make sense for a student's thesis, for a published article, it is inadequate. Hence, I would suggest compressing the manuscript by only pointing to the salient differences with respect to a reference combination.

**Response: We have compressed this section in the manuscript by excluding the repetition offered in the introductory portion of the results section and by also including: “See supplementary Figure 3 and Supplementary Table 1 for results obtained for MSD and HSD samples at different sequencing depths.” Instead of repeating the results for the said samples.**

5. Related to this, the groupings in Supplementary figures are different from those in main figures, i.e., grouped by variant p-distance instead of sequencing depth. This is of course acceptable but quite confusing for the reader. On the same point, legend of Figure 2 is likely incorrect: panel B should read LSD and not HSD. Finally, I would suggest using the authors' abbreviations here too: LSD_SV (instead of LSD alone) and LSD_DV (instead of HSD).

**Response: The grouping of figures in the supplementary figures has been adjusted to match that in the main text. Corrections noted have also been addressed.**

6. Back to manuscript length, I am doubtful about the usefulness of the first part of the results ("Global nucleotide comparison at different levels of contamination for different sequencing depths") and the associated heat maps. Which kind of insights and/or thresholds can be derived from this? Basically, these p-distances are already summarized in the phylogenetic trees, which are much more interesting as they further show the SNVs. That is why I would advise the authors to remove or considerably shorten this section.

**Response: We have significantly compressed this section as well. We have gotten rid of the repetition previously included in the introductory portion of this section of the results. We have chosen to retain the heatmaps as we feel it offers a quick visual comparison for those who do not have a good understanding or knowledge of how to read and interpret phylogenetic trees**.

7. The whole section of the discussion about mutational profiles (lines 385-404) should be transferred to the results because this where such a paragraph naturally belongs. This reorganization would also fix the lack of a proper introduction for Figure 4.

**Response: The suggested changes have been made. Lines 385 – 404 have been moved to lines 249 -266.**

8. Regarding Figure 5, it is quite large for a rather modest gain of insight. This is partly due to the choice of using pie-charts, which are known to have a very bad ratio of information content to chart size.

**Response: Figure 5 has been excluded.**

* Manuscript clarity

All the remaining comments are minor but should be addressed to improve the clarity of the manuscript. I list them below in line order.

- lines 95-97: "NGS runs are rarely repeated for reasons including limited funds to repeat expensive library preparation reactions and NGS remains relatively expensive, even when samples are multiplexed." Seemingly internally redundant sentence. Please rephrase.

**Response: The sentence has been rephrased to “NGS runs are rarely repeated for reasons including limited funds to repeat expensive library preparation reactions even when samples are multiplexed”**

- line 101: "the results of genomic analyses of organisms" I agree with the idea, but if this sentence concerns viruses, these are not organisms.

**Response: The sentence has been modified to include “the results of genomic analyses of organisms and viruses”.**

- lines 119 and following: It is difficult to understand where the empirical reads that are mixed up to generate artificial datasets come from. Are these from only four discrete samples among the 60+752 samples sequenced in the study? Please clarify this part of the design because it is confusing. Lines 172-173 are also related to this issue and should be clarified.

**Response: We apologize for the error- as stated, this should indeed be 60+752. We have simplified this passage in the text to better reflect that the ‘60+752’ known clinical sequences were evaluated to solely determine the number of reads to be used to generate the empirical reads. The empirical reads used for this study were obtained from mixing 4 ‘source’ genomes – known alpha, delta, and omicron sequences. To address the comment made, lines 118 -120 reflect the modifications made.**

- lines 126, 132, 175: "read length(s)" I do not see the point of mentioning read length in this context and I am pretty sure that at least at line 132, "length" should read "depth". Please check.

**Response: This has been amended.**

- line 128: "Random subsampled artificial sequences" Ambiguous wording that does not convey the idea of artificial FASTQ files mixing (subsampled) raw reads from different empirical samples. Please rephrase and do not use "sequences" for "reads" (also on line 348). On a related note, Figure 1 is not that clear because the idea of mixing reads is not explicitly represented.

**Response: The sentence in question has been corrected and Figure 1 has been amended to reflect the idea of mixing reads.**

- line 130: "15 different contamination levels" Correct but including no contamination at all, i.e., with background reads only. Please amend.

**Response: This has been amended.**

- line 152-153: "Reads were mapped to the reference 153 SARS-CoV-2 genome NCBI GenBank accession (MN908947)" For non-experts of SARS-CoV2 (and viruses in general), please explain why this step is needed and its role in calling the different types of SNVs discussed later (consensus SNVs vs variant SNVs).

**Response: An explanation has been provided for this. See lines 155 – 156.**

- line 159 (and elsewhere): "clinical samples" is ambiguous (see my other comments about details of the experimental design). Please clarify. Also, was there any ambiguity in these genuine "pure" samples? I think so if I look at Tables (e.g., 190 'N' for AY.25.1_low in Table 1). How so?

**Response: The reference genome sequence used for all the comparisons is the original Wuhan strain. We believe that it is crucial to keep this as the reference sequence for all comparisons since this is how ‘real’ SARS-CoV2 sequences are analyzed. As such, both the artificial samples and the ‘pure samples’ were compared to the Wuhan strain**.


- lines 161-162: "The p-distance option was chosen as input for the Model/Method setting while the default options were chosen for the other settings." Ok, but is it an observed distance or a model-corrected distance? Please specify.

**Response: Observed p-distance.**

- lines 185-186: "not substantial for contamination levels less than 20%" To me, it seems difficult to provide a single number for all the combinations (rather 20 to 40% depending on the case). Please check.

**Response: The sentence has been deleted**

- line 204 (legend of Figure 2): It would be useful to provide the p-distances between pairs of background strains in full numbers (not only in color shade).

**Response: Given the design of the figure/heatmap, including the p-distances between pairs of background strains in full numbers is not applicable. We have however enlarged the legend of the figure to give readers a better sense of the range of the squares**.

- lines 211-212: "by creating in silico artificial mixtures of reads to simulate contaminated genomes" I guess these mixtures were the same as before (section about Global comparison). If so, please rephrase because these look newly generated considering the introductory sentence.

**Response: We have deleted that portion of the manuscript as part of the effort to condense the length of the manuscript.**

- lines 221-222: For non-SARS experts, please explain the difference between consensus SNVs and variant SNVs (see my other comment on the issue above). I would have imagined that these SNVs are called against their respective background strain, but this does not look to be the case here.
**Response: We have defined consensus SNVs versus variant SNVs in the context of the manuscript. See lines 157-159.**

- line 225: "for all samples" appears useless and confusing. Please delete.

**Response: This has been amended.**

- lines 230-332: A verb is apparently missing in the sentence, e.g., "were observed". Please check.

**Response: This has been fixed**.

- line 247 (and other table legends): Most columns are not explained in the text and some are nor even used (e.g., lineage note, Scorpio call). Please check.

**Response: This has been amended. Columns not explained in the text have been excluded in the edited version of all tables.**

- line 306: "low sequencing depth sequence" should apply to both panels A and B, not only B. Please check.

**Response: This has been amended.**

- lines 321-324 (Figure 4): I fail to understand why some "mutations" are found across all strains and artificial samples. What was the reference genome?

**Response: The reference genome sequence used for all the comparisons is the original Wuhan strain. We feel it is important to keep this as the reference sequence for all comparisons since this is how ‘real’ SARS-CoV2 sequences are analyzed.**

- line 350: "for different sequences" I would have written "for both similar and different variants". Please check.

**Response: This has been amended to reflect the suggested comment.**

- lines 366-367: Please clarify the sentence because "lest" is confusing here. If it means "unless", it does not make sense in this context.

- lines 382-383 (and elsewhere): "genome integrity" I am not sure about this wording, which hints at some biological phenomenon. In my opinion, it is more a matter of "assembly accuracy" in this case.

**Response: This has been amended. "genome integrity" has been changed to “assembly accuracy” throughout the manuscript.**

- line 386: "the clinical samples and the artificially subsampled genomes" To me, the main variable is not the subsampling (because clinical samples are also subsampled) but rather the idea of artificial mixture". Please amend.

**Response: This has been amended to ‘artificially mixed’. Now Line 254**

- line 444: "linage" => lineage

**Response: This has been amended**.

Reviewer #2: In silico analyses identify sequence contamination thresholds for Nanopore-generated SARS-CoV-2 sequences

Synopsis:

The authors attempted to benchmark a method for determining the integrity of genomes and lineage calls of SARS-CoV-2 using ONT sequencing under contamination conditions of different viral strains. Systematic investigation of the effects of contamination on SARS-CoV-2 sequencing reads is sound, with the following recommendations:

Line 120: “Amplicons generated using tiling PCR”. Please explain why using amplicons. What primers were used?

**Response: Due to low quantities of viral genomic material in typical clinical specimens, the SARS-CoV-2 genomes must be amplified for high-throughput sequencing. This is commonly done by amplicon sequencing. This approach involves multiplexed PCR with numerous primer pairs tiled across the viral genome. We have omitted this explanation in the methods section as it will make this section cumbersome with details that do not necessarily add to the manuscript, full details of the approach are available in the published manuscript:** <https://www.ncbi.nlm.nih.gov/pmc/articles/PMC7480024/> .

Line 123: “60 SARS-CoV-2 samples sequenced on a MinION device and 752 samples sequenced on a GridION device”. Do they represent known Omicron, Delta and Alpha sequences used in the study?

**Response: We apologize for the lack of clarity - as stated in the response to Reviewer #1, this should indeed be 60+752 and the changes have been made intext. The authors have also clarified the origin of the empirical reads. The empirical reads used for this study were obtained from mixing 4 ‘source’ genomes – known alpha, delta, and omicron sequences. The ‘812’ known clinical sequences were evaluated to solely determine the number of reads to be used to generate the empirical reads.**

Line 130: “15 different contamination”. I counted 14 levels?

**Response: We have corrected this error.**

Line 132: “three read lengths”. Do you mean depths?

**Response: We have corrected this error.**

Line 154: “Pangolin (version 4.0.3, pangoLEARN) (version 1.2.333)”. Need a reference to cite authors.

**Response: We have included a citation for Pangolin.**

Line 155: “consensus sequences”. What criteria to call a consensus sequence? It forms an important point in your results.

**Response: This change has been made**.

Line 158: No phylogenetic analyses explained.

**Response: This was discussed in lines 365 - 375.**

Line 172: Can you tell the mean read length too?

**Response: We do not believe that including this information will offer any more insights. This is due to the nature of how the reads used in this study were generated. The generation of these reads was controlled from 4 ‘source/original’ genomes and so for each of the sequencing depths, the mean read lengths were consistent (see Figure 1).**

Line 173: “768 SARS-CoV-2 clinical samples”. It doesn’t add up for 60+752 samples in Line 123. Please explain.

**Response: This change has been made to reflect 60+752 samples. See line 118.**

Line 222: the number of consensus ‘n’. Please briefly explain how to call N in Methods, due to its importance in results. Is it 100% missing bp across all reads?

**Response: This change has been made. See Line 157.**

Line 345: Do you mislabel in Figure 5 between light and dark blue colors?

**Response: The figure has been deleted due to comments raised by another reviewer.**

Line 440: From “linage calls” to lineage calls.

**Response: This change has been made.**

Methods: Should also explain Scorpio call.

**Response: We have explained scorpio call -lines 160-162.**

Discussion: Please avoid repeating results and figures/tables in this section. Some might be better in the Results section.

**Response: We have reworked the manuscript the avoid repetition of results and figures/tables in the discussion.**

Discussion: The authors used consensus sequences, instead of de novo assembly. In my understanding, it is difficult to differentiate between contaminant and recombinant viruses. [Recombinant virus, please see “World Health Organization. 2022. TAG-VE statement on Omicron sublineages BQ.1 and XBB, 27 October 2022. [https://www.who.int/news/item/27-10-2022-tag-ve-statement-on-omicron-sublineages-bq.1-and-xbb](https://can01.safelinks.protection.outlook.com/?url=https%3A%2F%2Fwww.who.int%2Fnews%2Fitem%2F27-10-2022-tag-ve-statement-on-omicron-sublineages-bq.1-and-xbb&data=05%7C01%7Cayooluwa.bolaji%40phac-aspc.gc.ca%7Cd4d3950a577a4e33381e08dbeb9583cd%7C42fd9015de4d4223a368baeacab48927%7C0%7C0%7C638362799637265874%7CUnknown%7CTWFpbGZsb3d8eyJWIjoiMC4wLjAwMDAiLCJQIjoiV2luMzIiLCJBTiI6Ik1haWwiLCJXVCI6Mn0%3D%7C3000%7C%7C%7C&sdata=DUBV3OMoQbgdKdmQDFVLyhvS%2FF5mC2G9sjTi9E15%2Buw%3D&reserved=0).”] Although ONT long reads can define recombinants between viral strains using de novo assembly, this study doesn’t consider this to rule out recombination of a disease-causing virus from contaminant viruses. Please discuss this and provide future improvement.

If applicable, it would be nice to confirm truncated variants in Figure 4 and Figure S5 by other sequencing, especially illumina sequencing to see if the ONT error rates and corresponding pipeline can impact those variants between clinical and contaminant sequences.

**Response: While We appreciate the comment, we believe that it does not apply to the stated scope of this study, namely the identification of contamination thresholds for submission to public repositories**.

Should it be better to develop workflow to identify long reads with variants each representing different strains, instead of a consensus sequence of a single strain or artefactual hybrid strain? Should ONT reads need to be validated by using sanger sequencing due to its ONT bp accuracy?

**Response: We appreciate this comment and the insight this validation may provide. However, we believe that validating ONT reads with Sanger sequencing is not within the scope and objectives of this study.**

Discuss how to rule out multiple strain infection in a sample from a contamination event. Can it be co-infected with different strains within a sample?

**Response: The authors have addressed this from lines 388-392.**
