# Supplementary material for: In silico analyses identify sequence contamination thresholds for Nanopore-generated SARS-CoV-2 sequences
Source: PLoS Comput Biol. 2024 Aug 19;20(8):e1011539. doi: 10.1371/journal.pcbi.1011539 (PMC11398645; doi:10.1371/journal.pcbi.1011539)
Supplement: Attachment — Submitted filename: ResponseToReviewers_2024-05-31.docx [file pcbi.1011539.s009.docx]

Reviewer's Responses to Questions

Comments to the Authors:

Please note here if the review is uploaded as an attachment.

Reviewer #1: I have finally found some time to take a closer look at the revision. I apologize for the delay and I thank the authors for the changes they have made to address my previous comments. I think the revised manuscript is a huge step in the good direction but I still see a number of issues (some already raised in my original review) that should be taken care of before acceptance.

* "(not so) Minor issues"

1. Statistics are inexistant and this is annoying when trying to define thresholds. If I understand correctly, the authors have selected a number of metrics and the thresholds they eventually propose are the highest contamination proportions that do not affect any of these metrics in any analysis (i.e., across the two kinds of strain mixture and three sequencing depths). It makes sense as it is quite conservative, but still very empirical. Given the experimental design, I do not have a specific suggestion to improve this aspect, but I think I must underline it. For example for LSD_SV genomes around 5% contamination [lines 209-211 vs Table 2], most metrics are quite robust.

**Response: The metrics included in this study are those commonly used for the analysis of SARS-CoV-2 sequences in a clinical public health environment. We are maintaining the metrics used as the output for the ncov-tools pipeline (https://github.com/jts/ncov-tools), a pipeline used for the 700,000+ clinical samples sequenced as part of Canada’s genomic surveillance of SARS-CoV-2. We have not incorporated statistics in our analysis because it is incompatible with the structure of our study. We have an established pipeline, used by public health laboratories across Canada, we have the known (or true) sequence for the selected strains included in our study, and we have artificial mixtures of those strains. The purpose of this study isn’t to quantify the degree of effect of contamination but simply when the effect is measured, it is a binary variable in our study design – at what threshold of artificially induced contamination is there an observed effect on either the consensus sequence or the lineage call of the sample.**

2. I fail to understand how negative controls would work in practice. If a blank sample is contaminated by an non-targeted viral variant, the latter will occupy 100% of the sequencing reads. How to apply the identified thresholds then? Moreover, blanks can only control for one case of contamination (lab-introduced foreign sequences). Such an approach cannot control for samples contaminated at the time of collection (or at least before sample processing in the lab). Could the authors better explain the way they would use their thresholds to put away samples considered as too contaminated for deposit in public databases?

**Response: Indeed! We agree that the methods and conclusions made from this study address contamination generated during specimen processing and not at the collection stage. We believe addressing contamination at the collection stage is beyond the scope of this study hence no mention of that - one of the limitations of the study. Given the methods used in this study, we do not address contamination by non-SARS-CoV-2 viruses nor can we make conclusions about that. The aim of the study is to give researchers a baseline to determine if cross-contamination between samples has occurred this is because it is a common occurrence to observe mapped reads in any negative control and because of this it is (or should be) standard practice to quantify the number of mapped reads in the negative control.**

3. Some comments in the text do not seem to be compatible with the methods supposed to have been used. For example, about the global comparison and the heat map [lines 370-371], "The analysis performed considers all nucleotides present in the samples compared without any regard for the origin of the nucleotide (i.e., contaminant or not)." I am not sure about that: I understood that the authors compared the whole genomes assembled from the contaminated reads: [lines 159-160] "Aligned nucleotide consensus genome sequences of both the clinical samples and the artificially generated genomes were imported to MEGA11 software to calculate pairwise distance." With (only) 20% of contaminant reads, one can expect that the consensus assembled genome will nearly be the correct one, as indeed shown in the trees.

**Response: We have changed in text language in 2 places (lines 370-371 and lines 159-160). Indeed, with (only) 20% of contaminant reads one can expect that the consensus assembled genome will nearly be the correct one. However, the sentence/statement refers to the difference between observing a change at a single position, and observing a change in lineage call, averaged across 30,000 positions**

4. In Table 4, HSD_SV is correctly called for its lineage up to 50% contamination. I agree that it is what the results show in this case, but this does not make much sense. When two strains are mixed-up in equal amounts of reads, what should be the expected result? Here the lineage calling algorithm provides a result that apparently matches "the true strain" but there is no hard reason for this. It must be very dependant on the specific polymorphisms shared (or not) by the two closely related strains considered in the mixture.

**Response: In the scenario referenced by the reviewer, the two mixed strains are very. Both strains are delta sub-lineages (AY.25.1 and AY.27) with few differences as seen in S. Table 2A. There are undeniably complexities of the lineages calling algorithm, sublineages are defined by a variable number of mutations. But the nuances of the lineage definitions are beyond our control and scope; the Pangolin model is globally accepted for SARS-CoV-2 lineage definitions.**

* Clarity issues

- Figure 1 is useful to understand the experimental design but the mixing of viral strains could be better represented because there actually are only two combinations, not four, as possibly suggested by the figure.

**Response: We have modified Figure 1 to depict that 2 strains were mixed at any given time.**

- [lines 232-235] "(...) the number of consensus SNVs for the clinical omicron BA.1 sample was 56, the number of consensus ‘N’ as a measure of missing nucleotide was 189 and the number of variants SNVs was 61 (Table 3) when compared against the reference Wuhan strain (RefSeq NC_045512.2)." These results are more detailed than in the previous section, which does not make too much sense as they come second. At the opposite [lines 269-270] "assembly accuracy is maintained at contamination levels up to 6% for LSD (12,500 reads) and 7% for both MSD (25,000 reads) and HSD (50,000 reads) depths" has not been mentioned before this small conclusion, except as [lines 218-219] "See supplementary Figure 3 and Supplementary Table 1 for results obtained for MSD and HSD samples at different sequencing depths." Please try to balance the level of details throughout the text, i.e., avoid repeating the same result thrice whereas another important bit is only mentioned in a small conclusion.

**Response: We have addressed the issue of repetition.**

- [lines 247-248] "To identify the effect that artificial mixture had on both the clinical SARS-CoV-2 genomes and the artificially subsampled genomes" Why the "both"? When were the clinical genomes mixed-up in the experiments?

**Response: We have reworded this sentence. However, by investigating the effect of contamination on the artificially generated genome, conclusions can be made about the clinical genomes.**

- [lines 261-263] "(...) the artificially subsampled genomes that contained less than 6% of contaminant from a substrain of the same variant had similar mutational profiles to the clinical SARS-CoV-2 samples at all levels of contaminations." I don't understand the sentence: at <6% or at all levels?

**Response: At <6% or at all levels means - contamination levels from 1 to 5% . We have clarified this passage in the text.**

- [lines 264-266] "While the artificially generated subsampled genomes that contained less than 7% of contaminant from a divergent variant had similar mutational profiles to the corresponding clinical SARS266 CoV-2 samples at all levels of contamination." Same issue and no verb in the sentence.

**Response: We are unclear with this comment from the reviewer. This is not a stand-alone sentence - it is connected to the preceding sentence (Lines 262 - 264). The verb in this sentence is ‘had’ (to have)**.

- [line 353 and elsewhere] "artificially subsampled genomes" The authors use this wording in several places but, in my opinion, the main point is that these sample include contaminant reads. Thus, it would better read as "artificially subsampled and contaminated genomes". Similarly [line 194], "by subsampling the reads" should rather be "by subsampling and mixing the reads". Finally, in the same area of the text, as explained in my original review, these sentences are misleading because the discussed samples are in principle the same as those used in the previous section (about global comparison). Thus, their generation should have been explained before this section and not detailed here.

**Response: We have moved the sentence in question to lines 172 – 174. This allows it to be addressed earlier in the manuscript.**

- The legend for Figure S5 is missing. Moreover, please check the uniformity of the legends for all figures belonging to the same type of analysis (i.e., there exist three to six variants of each figure).

**Response: We have made adjustments to all Figures.**

The following phrases should be clarified too:

- [line 24] "maintain genomic sequence integrity"”

**Response: This has been adjusted to “maintain genome-relatedness and integrity was identified” - Line 24**

- [line 172] "The distance (proportion) of nucleotide sites"

**Response: This is to indicate that what was plotted was the p (proportion)-distance. This distance is merely the** proportion (p) of nucleotide sites at which the two sequences compared are different

[line 328] "lest" [already mentioned in my original review]

**Response: This is an appropriate use of the word ‘lest’. Lest can be used as a synonym for ‘for fear of’, ‘in case of’, ‘in order to avoid’, etc. In wordier language, our sentence is saying “…therefore it is critical that we maintain confidence in the sequences both submitted and pulled from public repositories in order to avoid erroneous variants affect major public health decisions.”**

- [line 556] "similar variants" [while the used code is HSD_DV] –

**Response: adjusted now line 578**.

* Other details

- [lines 208-209] "consensus ‘N’ (number of missing data sites)" Indeed, but I would rather say "undetermined" than "missing" because these sites are too polymorphic to be determined in the consensus genome due to contamination.

**Response: this has been adjusted**

- [line 128] "It is of note that in this study, the number of reads was used as a proxy for sequencing depth." This sentence can be safely deleted, since this precision is already mentioned a bit above [lines 99-101].

**Response: this has been adjusted**

- The following precisions could also be deleted:

- [line 23] "Through a series of analyses"

- [line 219] "at different sequencing depths"

- [lines 245-246] "at different sequencing depths"

- [line 255] "We believe that"

- [lines 287-288] "low sequencing depth sequence (12,500 reads)"

- Table 4 [head of column 2] "call" [it is incorrect]

**Response: The above phrases have been deleted.**

- The following typos should be fixed:

- [line 125] delete extra closing parenthesis

- [line 127] contaminate -> contaminating [or contaminant]

- [line 160] distance -> distances

- [line 176] composition negligible -> composition is negligible

- [line 199] served -> used

- [line 206] indel -> indels

- [line 207] call all -> call, all

- [line 212] levels -> level

- [lines 228 and 305] add missing closing parenthesis

- [line 401] practise -> practice

**Response: The above noted typos have been fixed.**

- Reviewer #2: Line 115, add this phrase at the beginning, “Due to low quantities of viral genomic materials in clinical specimens,”

**Response: This has been fixed.**

- Line 348, fix font size.

**Response: This has been fixed.**

-END-
